# Data-Driven Neurocognitive Clustering Predicts Virtual Reality Task Performance in Children: A Pilot Study

**DOI:** 10.3390/brainsci16050472

**Published:** 2026-04-28

**Authors:** Yumi Ju, Jihye Kim, Sura Kang, HyunJu Park

**Affiliations:** 1 Division of Occupational Therapy, Graduate School of Professional Therapy, Gachon University, Seongnam 13120, Republic of Korea; yumiju@gachon.ac.kr; 2 Center for Integrative Development & Psychology, Gachon University, Seongnam 13120, Republic of Korea; wisdom12391@gmail.com; 3 Convergence Research Center for Artificial Intelligence, Dongguk University, Seoul 04620, Republic of Korea; surakang7410@dongguk.edu; 4 Division of Speech-Language Pathology, Graduate School of Professional Therapy, Gachon University, Seongnam 13120, Republic of Korea

**Keywords:** developmental disability, theta/beta ratio (TBR), CANTAB, virtual reality, data-driven clustering, neurocognitive phenotype

## Abstract

**Highlights:**

**What are the main findings?**

- Data-driven clustering identified three neurocognitive profiles independent of diagnostic categories.
- Three neurocognitive profiles emerged: efficient, slow-accurate, and fast-error-prone (high TBR) differing in VR task errors.

**What are the implications of the main findings?**

- Transdiagnostic profiling better captures heterogeneity in cognitive processing.
- EEG and cognitive markers support individualized education and intervention planning.

**Abstract:**

**Background**: Traditional diagnosis-based classifications often fail to capture neurocognitive heterogeneity among children with developmental disabilities (DD). Establishing function-based subtyping is essential for developing individualized education frameworks that move beyond categorical labels. **Methods**: This pilot study employed a data-driven clustering approach integrating neurophysiological and cognitive indices to identify functional subtypes in 18 school-aged children (8 typically developing; 10 with DD). Input features included EEG-derived theta/beta ratio (TBR) and cognitive variables from the CANTAB Multitasking Test (MTT). Ecological validity was evaluated using the Virtual Kitchen Errand Task for Children (VKET-C). **Results**: K-means clustering revealed three distinct groups. In terms of MTT performance, Cluster 1 exhibited high accuracy and short response latencies. Cluster 2 demonstrated a “Slow but Accurate” pattern, with prolonged reaction times irrespective of diagnosis. Cluster 3 presented a “Fast but Error-prone” profile, showing significantly higher TBR values and increased error rates, indicative of cognitive impulsivity. Notably, clusters did not align with diagnostic boundaries. The three identified clusters significantly differentiated commission errors on the VKET-C task and showed greater explanatory power for VR task performance than diagnosis-based classifications. **Conclusions**: Cluster-based classification better differentiated VR task performance, particularly commission errors, than traditional diagnosis-based grouping. Integrating diagnosis with neurocognitive deep phenotyping approaches may enable more individualized intervention and educational support for children.

## 1. Introduction

Developmental disability (DD) including Autism Spectrum Disorder (ASD) and Intellectual Disability (ID) encompasses a wide range of cognitive and behavioral characteristics, often marked by significant heterogeneity among individuals [1]. Despite this variability, most clinical and research practices continue to rely on diagnosis-based comparisons between children with DD and typically developing (TD) peers. Such binary classifications may not adequately reflect actual differences in functional performance or the level of support needed by individual children [2,3]. Diagnostic status alone may be insufficient to capture the full spectrum of occupational functioning in children. Accordingly, it is essential to establish research frameworks that move beyond categorical diagnoses and embrace the multidimensional nature of functional cognitive performance in daily tasks.

To address these limitations, researchers have increasingly emphasized multidimensional function-based subtyping rather than relying on broad diagnostic categories [4]. A data-driven clustering approach that integrates cognitive and neurophysiological data beyond diagnostic boundaries can help delineate the heterogeneity embedded within diagnostic labels [5]. This approach offers a more nuanced understanding of functional diversity and provides a foundation for individualized intervention planning in children with DD.

Among the multiple dimensions of developmental functioning, cognitive ability has been consistently identified as a key predictor of adaptive behavior, influencing children’s adjustment in daily and school contexts [6]. However, cognitive measures alone may not fully reflect the neural underpinnings of individual differences in functional performance. Neurophysiological indices derived from electroencephalography (EEG) can complement cognitive assessments by providing objective evidence of mental workload, fatigue, and mental effort [7]. One commonly used EEG- derived index is the theta/beta ratio (TBR), a measure derived from resting-state EEG that has been linked to cognitive load, attentional regulation, and executive functioning [8,9]. When combined with behavioral performance indicators such as error profiles, TBR may serve as an integrative biomarker for identifying functionally meaningful subtypes within the heterogeneous DD population.

The advantage of function-based clustering that integrates cognitive and neurophysiological measures lies in its capacity to move beyond the conventional dichotomy of simply “good” or “poor” performers. Rather than focusing solely on outcome-based distinctions, this approach facilitates the identification of distinct processing characteristics that reflect both neural and behavioral dimensions of functioning [5]. Such an approach can reveal children who, despite being typically developing, exhibit reduced efficiency in cognitive processing [10]. They may achieve accurate performance but require greater cognitive effort or mental load, resulting in slower processing speed [11]. Conversely, there are also children diagnosed with developmental disabilities who demonstrate relatively well-preserved or high functioning cognitive profiles [12]. For clinicians and educators seeking to deliver individualized therapeutic support, evaluating performance through the lens of processing efficiency rather than absolute task success provides a more meaningful framework for identifying children who may benefit from intervention.

While data-driven clustering based on cognitive and neurophysiological indicators can reveal latent functional subtypes, an important question remains as to whether these clusters correspond to meaningful differences in real-world performance. In other words, it is crucial to determine whether the identified subgroups not only differ in their neural or cognitive profiles but also in how they carry out goal-directed tasks that reflect everyday functioning. Addressing this question requires an ecologically valid assessment context that engages integrated executive processes such as planning, sequencing, and self-monitoring. An increasing number of studies have demonstrated the usefulness of virtual reality (VR) in assessing cognitive functions in individuals with neurodevelopmental disorders [13,14]. This growing body of evidence highlights the potential of VR as a clinically relevant assessment tool, particularly for capturing cognitive performance in ecologically valid contexts. In this regard, Virtual Reality (VR)-based performance tasks offer a promising approach, as they simulate complex, naturalistic environments while preserving experimental control [15,16]. Such tasks enable the observation of performance errors and behavioral efficiency under conditions that approximate real-life demands, thereby bridging the gap between laboratory-based measures and everyday functional outcomes.

Therefore, this pilot study aimed to identify functionally distinct subgroups of children using a data-driven clustering approach that integrated neurophysiological and cognitive performance indices. We further examined whether these clusters exhibited differential patterns of ecological task performance in a VR kitchen task. By linking neurocognitive profiles to ecologically valid behavioral outcomes, this study aimed to clarify how variations in processing efficiency are reflected in everyday functional performance.

## 2. Methods

### 2.1. Participants

A total of 18 school-aged children participated in this study, including 8 typically developing children and 10 children with developmental disabilities. Children with DD were defined as those with an IQ below 70 who were classified as eligible for Special Education Needs (SEN). Exclusion criteria included children with impaired mobility posing a risk of falls and those with visual impairments. Among the children with DD, 9 were children with ID (mild) and 1 was a child with ASD. The mean age of the participants was 9.72 years (SD = 1.6, Range 7–12), and the sample consisted of 10 boys and 8 girls. Participants were recruited from several schools in the local community. Written informed consent was obtained from the parents or legal guardians of all participants prior to participation. The study protocol was reviewed and approved by the Institutional Review Board (IRB) of Dongguk University (DUIRB-20231026).

### 2.2. Equipment

(1) CANTAB

**Figure 1:**
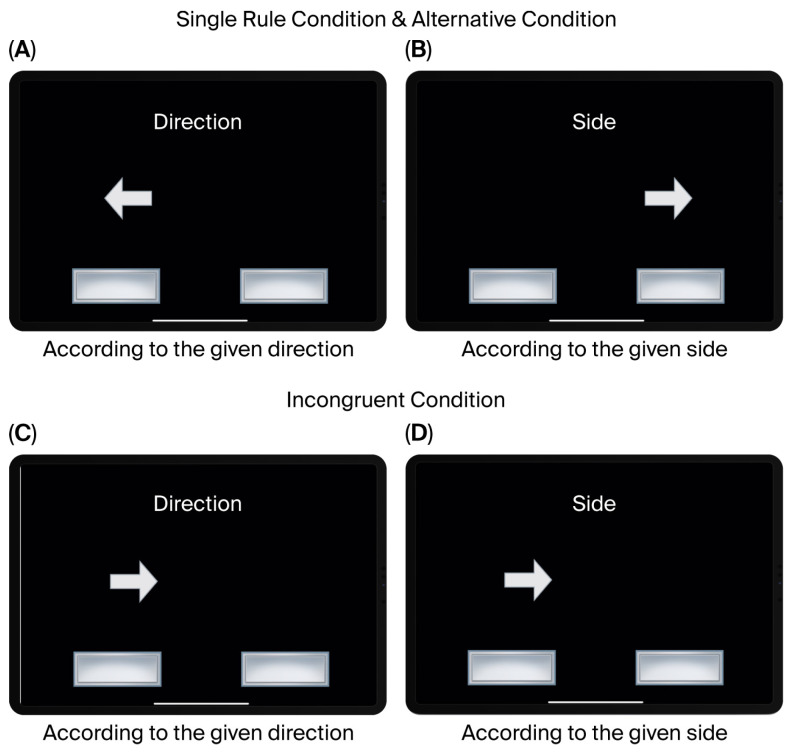
Multitasking Test (MTT). (**A**,**B**) Single-task and alternating conditions requiring responses based on stimulus direction (of the arrows) or side (of the rectangle). In the single condition, rules were presented separately; in the alternative condition, rules alternated across trials. (**C**,**D**) Incongruent trials in which direction and side were mismatched, requiring rule-based responses despite conflict.

Multitasking Test (MTT) from the Cambridge Neuropsychological Test Automated Battery (CANTAB, UK) was administered to assess executive function in children. The MTT is a computerized neuropsychological task designed to evaluate attention, cognitive flexibility, and inhibitory control. The MTT has been used in pediatric populations, including children with developmental disorders such as ADHD, ASD, and ID. The task consists of three conditions: (1) a single-task condition, in which responses are made according to a single rule, (2) a mixed-task condition, where different rules are applied alternately, and (3) an incongruent condition, in which conflicting information is presented (Figure 1). From these conditions, quantitative performance indices such as reaction time, accuracy, task-switching cost, and interference effect were derived (Table 1).

(2) EEG

**Figure 2:**
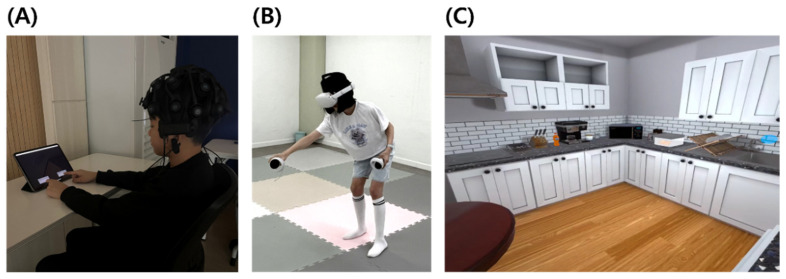
Experimental setup and task environments. (**A**) EEG recording during administration of the CANTAB cognitive tasks. (**B**) Participant performing the VR kitchen task. (**C**) Screenshot of the VR kitchen environment.

Electroencephalographic (EEG) data were recorded using the Cognionics CGX-Quick 20r wireless system (Cognionics Inc., San Diego, CA, USA), which employs 20 channels arranged according to the international 10–20 electrode placement system. Raw EEG data were preprocessed using standard procedures by using EEGLAB. The sampling rate was set at 256 Hz, and impedance levels were maintained below 50 kΩ, as recommended for this system. Data were band-pass filtered between 0.5 and 40 Hz and notch filtered at 60 Hz to remove power line interference. Data were cleaned for artifacts using ICA (Independent Component Analysis) and artifact-contaminated segments exceeding 100 μV were excluded. EEG signals were continuously recorded during the experimental task. Recordings were conducted in a quiet, dimly lit room to minimize environmental noise and distractions (Figure 2A).

Theta/Beta Ratio (TBR) was computed as the mean theta power (4–7 Hz) divided by the mean beta power (13–30 Hz) from frontal electrodes. TBR is widely used as an index of attentional regulation and cortical arousal, and elevated ratios are often interpreted as reflecting inattention or executive control difficulties in developmental populations [17,18].

(3) Virtual Kitchen Task

The Virtual Kitchen Errand Task for Children (VKET-C) was designed to assess functional cognition using HMD-based VR (Oculus Quest2, CA, USA) [16]. Participants completed three sequential actions: placing bread on a plate, pouring coffee into a mug, and retrieving a banana from the refrigerator to set all items on the table (Figure 2B,C). The task required working memory and executive functions for efficient sequencing. Prior to testing, participants practiced using the controller and were instructed to perform as quickly and accurately as possible. Task performance errors were classified as omission, commission, sequence errors (Table 2). Two occupational therapists independently reviewed video recordings and reached consensus through repeated observation.

### 2.3. Statistical Analysis

Cluster analysis was performed using Python 3.10. The k-means algorithm was applied, and the optimal number of clusters was determined as K = 3 based on the elbow method. For the clustering procedure, the TBR indices obtained from the three conditions of the MTT task and ten cognitive outcome variables from the MTT of CANTAB battery were included as input features. The differences in virtual task (VKET-C) performance errors among the clusters were statistically tested using one-way analysis of variance (ANOVA). Statistical significance was set at *p* < 0.05. Prior to analysis, all variables were standardized to ensure comparability across different measurement scales.

## 3. Results

### 3.1. Cluster Characteistics Based on EEG and MTT Performance

Three clusters were identified based on TBR indices across task conditions and MTT performance. Cluster 3 showed consistently higher TBR values across all conditions compared to Clusters 1 and 2.

In terms of MTT performance, Cluster 3 exhibited significantly higher error rates across multiple indices (CE, ICE, STBE, and MTBE), while consistently showing the shortest response latencies. In contrast, Cluster 2 was characterized by prolonged response times across several latency indices (LCM, LNOM, LMTM, and LSTM). Additionally, it demonstrated greater response delays under cognitively demanding conditions, as indicated by higher ICOST and MTCM values. For the last, Cluster 1 showed lower error rates and faster response times than the other clusters.

Descriptive statistics of the neurophysiological and cognitive variables across clusters are presented in Table 3.

### 3.2. PCA Visualization of Cluster Distribution

**Figure 3:**
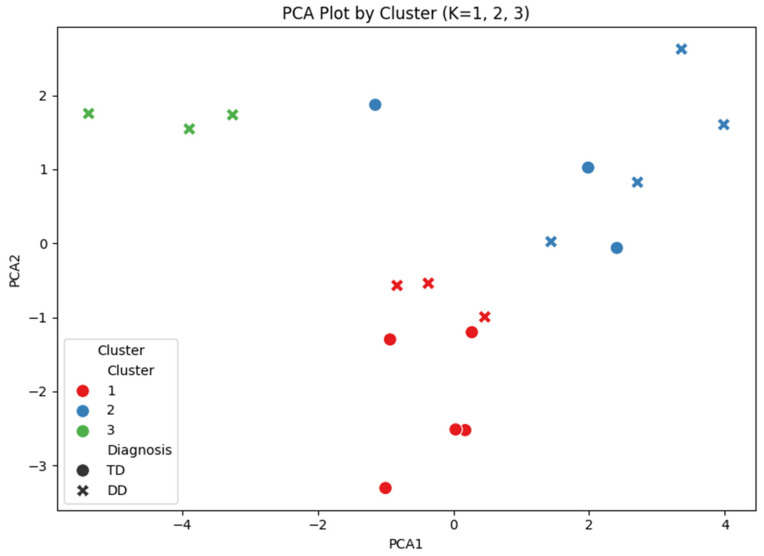
PCA Plot with Three Clusters.

**Figure 4:**
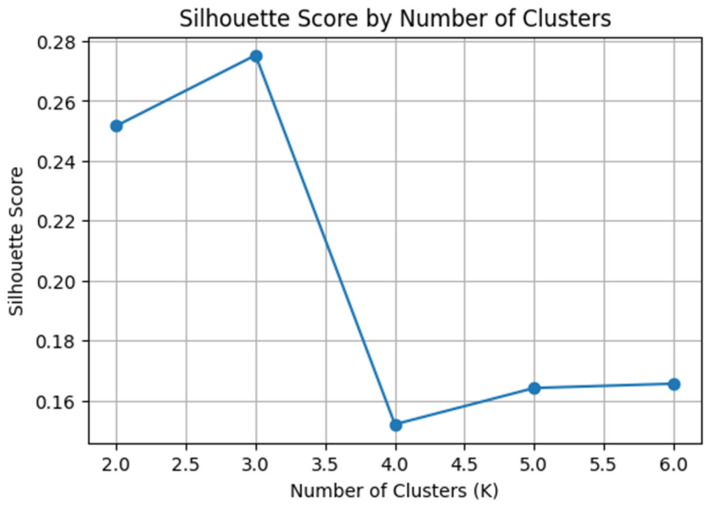
Determination of the optimal number of clusters (K) using Silhouette coefficients.

Visual inspection of the PCA plot revealed discernible clustering patterns among the three groups (Figure 3). The Silhouette coefficient reached its peak at K = 3 (0.275), followed by a sharp decline from K = 4 onwards (Figure 4). Similarly, the Dunn Index showed its optimal value (0.236) at K = 3, confirming the consistency between the two validity indices. Despite the modest Silhouette score inherent in high-dimensional pilot datasets, K = 3 was selected as the most robust and interpretable model.

**Figure 5:**
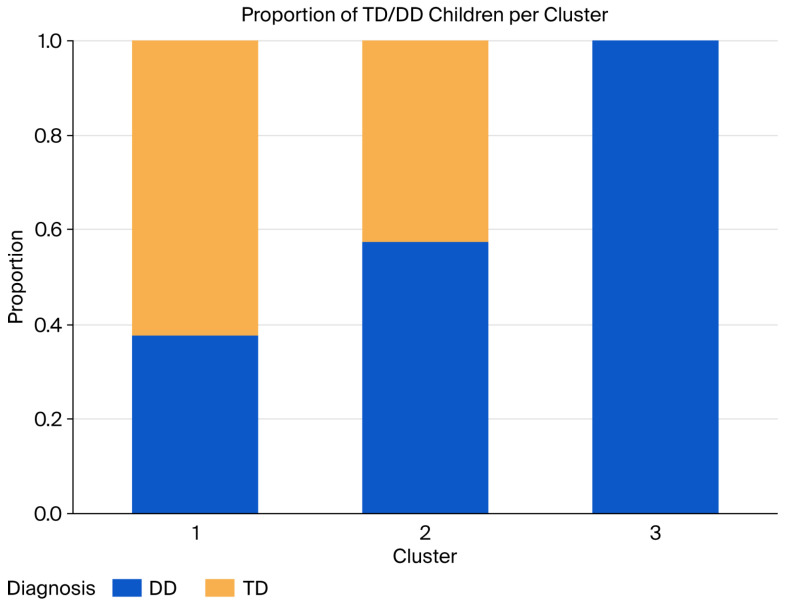
Proportion of TD and DD children in each cluster.

Examination of group composition further indicated that both TD and DD children were present across clusters. Cluster 1 comprised 5 TD (62.5%) and 3 DD children (37.5%). In Cluster 2, TD accounted for 3 (42.9%), whereas DD comprised 4 (57.1%). Cluster 3 consisted entirely of DD children (*n* = 3, 100%) (Figure 5).

### 3.3. Differences in VR Task Performance Across Clusters

To examine differences in VR task performance, a series of analyses were conducted to compare the explanatory power of the data-driven clusters versus traditional diagnostic categories (Table 4).

First, ANOVA revealed that the cluster-based classification significantly differentiated performance patterns, particularly for commission errors (*F*(2,15) = 4.875, *p* = 0.023, *η*^2^ = 0.393). Although differences in omission errors approached the threshold, they did not reach statistical significance (*F*(2,15) = 3.671, *p* = 0.05) and no significant differences were found for sequence errors (*F*(2,15) = 1.193, *p* = 0.33).

Importantly, when comparing these results to the traditional diagnostic groups (TD vs. DD) using independent *t*-tests, the diagnostic model failed to show any significant differences across all error types (*p* > 0.05). A comparison of effect sizes further demonstrated that the cluster-based model consistently captured a substantially larger portion of the variance in performance compared to the diagnostic model. Notably, for commission errors, the cluster model explained 39.3% of the variance (*η*^2^ = 0.393), whereas the diagnostic model explained only 1.9% (*η*^2^ = 0.019).

Post-hoc analyses revealed no significant differences in omission errors between clusters, except for a significant difference between Cluster 1 and Cluster 3 (*p* = 0.043). For commission errors, Cluster 3 showed significantly higher error rates compared to both Cluster 1 (*p* = 0.020) and Cluster 2 (*p* = 0.047), whereas no difference was observed between Cluster 1 and Cluster 2. No significant differences were found across clusters for sequence errors (Table 5).

## 4. Discussion

The present study applied data-driven clustering that integrated cognitive and neurophysiological indicators to identify functional subtypes among children with developmental disabilities (DD) and typically developing (TD) children, and further examined how these subtypes were associated with performance in a VR-based daily living task. Three distinct functional clusters emerged, differentiated by cognitive processing speed, accuracy, and electroencephalographic indices (TBR).

Notably, the derived clusters did not align neatly with traditional diagnostic boundaries (TD vs. DD), as both TD and DD children were distributed across clusters. For example, a substantial proportion of TD children were included in Cluster 2, characterized by cognitive inefficiency, whereas some DD children were classified into Cluster 1, showing relatively efficient behavioral performance. These patterns suggest that behavioral performance in ecologically valid tasks may not be fully explained by diagnostic status alone [19,20].

It is noted that 3 clusters demonstrated significant differences in commission error pattern, but not in omission and sequence errors during the VR kitchen task. Commission errors sensitively reflect inhibitory control capacities [21], which tend to exhibit substantial inter-individual heterogeneity. Notably, statistical significance was observed exclusively in commission errors, suggesting that our neurocognitive cluster-based classification effectively captured underlying neurocognitive heterogeneity that remains obscured in traditional diagnostic categories. Furthermore, the cluster model accounted for 39.3% of the variance (*η*^2^ = 0.393), compared to 1.9% (*η*^2^ = 0.019) in the diagnostic model. It means that the cluster-based approach demonstrated greater sensitivity in differentiating children who exhibited elevated commission errors, indicating its potential advantage in identifying inhibitory control deficits.

However, these findings should be interpreted with caution, as the cluster-based classification demonstrated statistically meaningful separation primarily within the VR task performance. This suggests that the identified clusters may be most sensitive to task-specific behavioral expressions rather than broadly generalizable functional distinctions. Thus, rather than replacing diagnostic frameworks, integrating them with deep phenotyping approaches may provide a more comprehensive understanding of individual differences [22].

Omission errors are typically considered to reflect deficits in sustained attention [23]. However, such errors are more likely to emerge under conditions of reduced engagement or increased attentional demand. In the present study, the immersive and interactive nature of the VR-based task may have enhanced task engagement, thereby supporting sustained attention and reducing variability in omission errors across participants [24]. Although post-hoc analyses revealed a significant difference between Cluster 1 and Cluster 3, no significant differences were observed between adjacent clusters (Cluster 1 vs. Cluster 2, Cluster 2 vs. Cluster 3). This pattern suggests that omission errors may be sensitive to more pronounced differences in attentional functioning but have limited sensitivity in capturing more subtle, fine-grained distinctions between neurocognitive profiles.

While sequence measures are intended to capture adherence to a logical action order, they may also reflect the efficiency or organization of task performance. In the present study, the relatively low sequencing demands of the task may have limited its sensitivity to higher-order deficits in action planning and organization. Moreover, in everyday task contexts, deviations in action sequence do not necessarily indicate illogical performance but may instead reflect individual differences in task organization or strategy [25]. Therefore, sequencing measures may have limited sensitivity in capturing differences between clusters defined by neurocognitive characteristics.

Examining cluster-specific characteristics further, Cluster 3 demonstrated the shortest latency yet the highest error rates (CE, ICE, STBE, MTBE). This “Fast but Error-prone” performance pattern is indicative of cognitive impulsivity. The elevated TBR observed in this cluster provides neurophysiological support for this interpretation. Prior research has shown that increased TBR reflects diminished inhibitory control associated with frontal lobe immaturity, resulting in reduced attentional regulation during cognitive processing [26]. Taddeini [27] further reported that when pre-response processing in the prefrontal cortex is bypassed under high cognitive load, fast-automatic responses tend to dominate. Accordingly, the heightened TBR in Cluster 3 suggests that motor responses were executed impulsively before stimuli were adequately evaluated. Thus, the elevated error rate observed in this cluster may not reflect a simple deficit in ability but rather a failure of cortical inhibitory control mechanisms during high-demand cognitive processing [28].

In contrast, Cluster 2 exhibited the longest reaction times while maintaining accuracy comparable to Cluster 1, reflecting a “Slow but Accurate” cognitive style characterized by deliberate processing and compensatory control [29]. This pattern suggests that increased cognitive effort, potentially supported by heightened beta activation, is recruited to preserve accuracy at the expense of processing speed. Consistent with the “High accuracy—Slow RT” profile observed in ADHD-I subtypes [30], these children may rely on resource-intensive strategies to achieve normative performance. From an educational perspective, this profile indicates heightened vulnerability under time pressure or high cognitive load; however, with appropriate scaffolding and sufficient processing time, their functional performance and academic potential may be effectively supported.

Beyond theoretical interpretation, the present findings have direct implications for clinical and educational practice. First, the identification of neurocognitive subtypes provides a framework for individualized intervention planning [31]. For example, children in the “Fast but Error-prone” cluster may benefit from interventions targeting inhibitory control and response monitoring, such as metacognitive strategies and structured feedback. In contrast, children in the “Slow but Accurate” cluster may require environmental adaptations that reduce time pressure and cognitive load, allowing sufficient processing time to maintain accuracy.

Second, the VR-based assessment paradigm (VKET-C) demonstrates feasibility as an ecologically valid tool for evaluating functional cognition in everyday-like contexts. Unlike traditional laboratory-based cognitive tests, this approach captures real-time performance errors, providing actionable insights for practitioners [32]. With advances in portable VR systems and automated data logging, such assessments may be increasingly implementable in school and clinical settings with minimal disruption to routine practice. Furthermore, the integration of data-driven clustering with performance-based VR assessment offers a scalable decision-support framework for therapists and educators, enabling targeted intervention planning and ongoing monitoring of functional outcomes.

Nevertheless, several limitations should be acknowledged. The relatively small sample size may limit the stability and generalizability of the clustering solution; therefore, replication with larger and more diverse cohorts is necessary to confirm the robustness of the identified functional profiles.

In addition, although TBR was interpreted within established theoretical frameworks of inhibitory control and attentional regulation, reliance on a single neurophysiological index may not fully capture the complexity of the underlying cortical mechanisms. Accordingly, incorporating multimodal neurophysiological measures and longitudinal designs in future research would enable a more comprehensive understanding of how these cognitive–neural profiles develop and relate to functional outcomes over time.

Taken together, these limitations highlight the need for cautious interpretation while also underscoring directions for future research. Despite these constraints, the present study contributes to the growing body of research on integrating neurophysiological and cognitive–behavioral measures to better understand developmental heterogeneity. The findings suggest that data-driven, deep phenotyping approaches may provide complementary insights beyond traditional diagnostic frameworks. Further research with larger samples is warranted to clarify the stability and practical implications of these subgroup patterns. Ultimately, this approach may support more precise characterization of individual neurocognitive profiles and inform targeted, functionally relevant intervention strategies.

## 5. Conclusions

This study aimed to identify neurocognitive heterogeneity across children with typical development and those with developmental disabilities. Cluster analysis was conducted to derive functional subgroups, resulting in three distinct clusters characterized by differing cognitive and neurophysiological profiles: fast and accurate, slow but accurate, and fast but error-prone with elevated impulsivity reflected in EEG indices. These clusters showed significant differences in commission errors during the Virtual Kitchen Task (VKET-C), and demonstrated greater explanatory power for VR task performance than traditional diagnosis-based classifications. This finding suggests that a cluster-based approach may offer enhanced sensitivity in identifying children with elevated commission errors, reflecting underlying deficits in inhibitory control. Integrating diagnosis with neurocognitive deep phenotyping approach may therefore facilitate more precise, individualized intervention and educational support for children.

## Figures and Tables

**Table 1:** Definition of variables on MTT of CANTAB.

Sub-Item Title	Description
Congruent Errors (CE)	The number of assessed congruent trials for which the trial outcome was an incorrect response (subject pressed the wrong button).
Incongruent Errors (ICE)	The number of assessed incongruent trials for which the trial outcome was an incorrect response (subject pressed the wrong button).
Single Task Block Errors (STBE)	The number of trials in assessed block(s) in which only a single rule is used, and the trial outcome was an incorrect response.
Multitasking Block Errors (MTBE)	The number of trials in assessed block(s) in which both rules are used, and the trial outcome was an incorrect response.
Reaction Latency Mean-Congruent (LCM)	The mean latency of response (from stimulus appearance to button press) on congruent trials in all assessed blocks.
Reaction Latency Mean-Incongruent (LNOM)	The mean latency of response (from stimulus appearance to button press) on incongruent trials in all assessed blocks
Reaction Latency Mean—Multitasking Blocks (LMTM)	The mean latency of response (from stimulus appearance to button press) in assessed block(s) in which both rules are used.
Reaction Latency Mean—Single Task Blocks (LSTM)	The mean latency of response (from stimulus appearance to button press) in assessed block(s) in which only one rule is used.
Incongruency Cost (ICOST)	The difference in mean response latency between congruent and incongruent trials; positive values indicate faster responses on congruent trials, with higher incongruency costs reflecting slower processing of conflicting information.
Multitasking Cost (MTCM)	The difference in mean response latency between multitasking blocks (both rules) and single-rule blocks; positive values indicate slower responses during multitasking, reflecting a higher cost of managing multiple sources of information.

**Table 2:** Performance Error Type and Definition in Virtual Kitchen Task.

Error Type	Description
Omission	Omission errors are operationally defined as the absence of necessary actions or steps that are required to complete a task.
Commission	Unnecessary actions are defined as extraneous behaviors, including tool misuse, redundant repetitions, or touching objects without purpose.
Sequence Error	Deviations from the intended order of steps, resulting in inefficient or incorrect task execution.

**Table 3:** Neurophysiological and Cognitive Variables in EEG and CANTAB in Each Cluster.

Variables		Cluster 1	Cluster 2	Cluster 3
TBR	Condition 1	8.4 (5.12)	8.43 (7.67)	33.37 (42.82)
	Condition 2	8.57 (3.62)	9.28 (6.57)	17.53 (9.26)
	Condition 3	11.2 (10.3)	10.38 (10.39)	13.41 (12.61)
MTT	CE	0.88 (0.83)	0.14 (0.38)	14 (8.72)
	ICE	9.25 (3.77)	11.57 (11.65)	28.67 (3.06)
	STBE	3 (1.6)	4.86 (7.8)	19.33 (7.37)
	MTBE	7.12 (4.76)	6.86 (4.53)	23.33 (2.89)
	LCM	642.95 (73.16)	855.84 (109.07)	637.08 (68.73)
	LNOM	661.69 (73.66)	986.41 (0.47)	641.79 (59.48)
	LMTM	729.59 (76.63)	1043.89 (155.26)	574.97 (103.01)
	LSTM	575.13 (100.17)	802.58 (72.37)	706.64 (24.7)
	ICOST	18.74 (29.16)	130.57 (93.14)	4.71 (53.76)
	MTCM	154.46 (105.19)	241.31 (101.14)	−131.68 (95.98)

**Table 4:** ANOVA of VR Performance Error Frequency by Cluster.

Cluster -based		Mean Frequency				*F* (*df*1, *df*2)	*p*-value	*η* ^2^
		Cluster 1	Cluster 2		Cluster 3			
	Omission	0.25 (0.71)	0.57 (0.79)		2.33 (2.52)	3.671 (2,15)	0.050	0.328
	Commission	2.88 (2.3)	3.86 (3.8)		10.67 (6.66)	4.875 (2,15)	0.023 *	0.393
	Sequence	2.50 (2)	2.00 (1.15)		3.67 (0.58)	1.193 (2,15)	0.330	0.137
Diagnosis -based		Mean Frequency				*t* (*df*)	*p*-value	*η* ^2^
		TD		DD				
	Omission	0.25		1.10		1.395	0.182	0.108
	Commission	3.87		5.10		0.557	0.585	0.019
	Sequence	3.12		2.00		−1.562	0.138	0.132

* *p* < 0.05.

**Table 5:** Post-hoc Analysis of Commission Errors Across Clusters.

Error Types	Comparison	Mean Diff	*p*-Adjusted	95% CI
Omission	Cluster 1 vs. Cluster 2	0.32	0.853	−1.22, 1.86
	Cluster 1 vs. Cluster 3	2.08	0.043 *	0.05, 4.10
	Cluster 2 vs. Cluster 3	1.76	0.100	−0.30, 3.82
Commission	Cluster 1 vs. Cluster 2	0.98	0.870	−4.08, 6.04
	Cluster 1 vs. Cluster 3	7.79	0.020 *	1.17, 14.41
	Cluster 2 vs. Cluster 3	6.81	0.047 *	0.06, 13.56
Sequence	Cluster 1 vs. Cluster 2	−0.50	0.812	−2.60, 1.60
	Cluster 1 vs. Cluster 3	1.16	0.527	−1.58, 3.91
	Cluster 2 vs. Cluster 3	1.66	0.299	−1.13, 4.46

* *p* < 0.05, Mean Diff = Mean Difference, CI = Confidence Interval.

## Data Availability

Data are available from the DGU Institutional Data Access/Ethics Committee (contact via Yumi Ju (yumiju@gachon.ac.kr) for researchers who meet the criteria for access to confidential data).
